# Transcription Factor Regulation of Epidermal Differentiation and Inflammation

**DOI:** 10.3390/cells15161479

**Published:** 2026-08-18

**Authors:** Uyanga Batzorig, Grace Zhang, George L. Sen

**Affiliations:** Department of Dermatology, Department of Cellular and Molecular Medicine, UCSD Stem Cell Program, University of California, San Diego, La Jolla, CA 92093, USA; ubatzorig@health.ucsd.edu (U.B.); gzhang5903@gmail.com (G.Z.)

**Keywords:** transcription factors, ZNF750, OVOL1, GRHL3, skin barrier, skin inflammation, psoriasis, atopic dermatitis

## Abstract

The epidermis is far more than a mechanical shield; it is a dynamic immunological interface whose structural integrity and immune homeostasis are co-regulated by interconnected transcriptional networks. This review examines how epidermal differentiation promoting transcription factors coordinate terminal keratinocyte maturation, skin barrier formation, immune suppression, lipid metabolism, and tissue repair. In particular, Zinc finger protein 750 (ZNF750), grainyhead-like transcription factor 3 (GRHL3), and ovo-like transcriptional repressor 1 (OVOL1) integrate epidermal differentiation with the suppression of inflammatory signaling by regulating lipid metabolism, innate immune sensors, stress-response pathways, and environmentally responsive transcriptional programs. Disruption of these regulatory nodes impairs barrier integrity, amplifies inflammatory signaling, and predisposes to chronic skin diseases, including psoriasis and atopic dermatitis.

## 1. Introduction

The skin is the largest organ in the human body, serving as the primary physical barrier that shields the body against pathogens, ultraviolet (UV) radiation, extreme temperatures, and water loss [1,2]. This protective role relies on the structural integrity of the epidermis, the outermost skin layer, which develops from the ectoderm via several stages: the periderm, germinative layer, and intermediate layer. As it matures, keratin expression transitions from keratins 8 and 18 (K8/K18) in the periderm to keratin 5 and 14 (K5/K14) in the basal layer, and keratins 1 and 10 (K1/K10) in the spinous layer. Markers of terminal differentiation, such as filaggrin (FLG) and loricrin (LOR), help form the cornified envelope [3,4,5]. This envelope creates a physical barrier that prevents water loss through the skin and blocks pathogens, crucial for survival after birth and maintaining skin health over time [6,7]. The process of epidermal differentiation is highly coordinated and regulated by complex mechanisms, including epigenetic modifications, transcription factor networks, signaling pathways, and post-transcriptional controls [5,8,9,10].

These mechanisms operate hierarchically: master regulators such as tumor protein P63 (p63) are necessary for acquisition of epidermal identity, while downstream terminal regulators, ZNF750, Kruppel-like factor 4 (KLF4), GRHL3, and OVOL1, execute the differentiation program by activating structural barrier genes including *FLG*, *LOR*, Claudin 1(*CLDN1*), Aquaporin 3 (*AQP3*), involucrin (*IVL*), Calmodulin-like 5 (*CALML5*), and repressing proliferative programs [7,9,11,12,13,14]. Dysregulation of any of these transcription factors within this hierarchy impairs cornified envelope assembly, alters lipid metabolism, and compromises barrier competence, predisposing the skin to inflammatory disease, delayed wound healing, and epithelial malignancy [15].

Importantly, the epidermis functions as far more than a passive mechanical shield [16]. Keratinocytes engage both innate and adaptive immune pathways via cytokine and chemokine secretion, pattern recognition receptor expression, and antigen presentation, positioning the epidermis as a dynamic immunological interface [17,18,19,20]. Epidermal differentiation transcription factors such as ZNF750, GRHL3, and OVOl1 are integral to this immunological function: in addition to orchestrating structural barrier assembly, they constitutively suppress inflammatory gene programs, thereby restraining cutaneous immune activation under homeostatic conditions. This allows the outermost differentiated layers of the epidermis not to overreact to inflammatory stimuli present on the surface of the skin. When these factors are lost or functionally compromised by mutation or haploinsufficiency, barrier and immune dysregulation occur together, leading to the chronic inflammation characteristic of diseases such as psoriasis and atopic dermatitis.

Although epidermal differentiation and cutaneous immune regulation have traditionally been regarded as separate biological processes, mounting evidence suggests that they are interconnected through shared transcriptional networks. An increasing number of differentiation-associated transcription factors concurrently regulate structural barrier formation, lipid metabolism, stress responses, and inflammatory signaling. This review will focus on 3 differentiation promoting transcription factors (ZNF750, GRHL3, and OVOL1) that have been shown to directly suppress the inflammatory response. ZNF750, GRHL3, and OVOL1 were chosen since there is supporting evidence for (1) epidermis gain or loss of function phenotype with inflammatory consequence; (2) direct binding of these factors to inflammatory genes which leads to their suppression. While other factors such as P63, activator protein 1 (AP-1) and CCAAT/enhancer-binding protein (C/EBP) family members, NOTCH signaling pathway, and KLF4 have been shown to promote the differentiation process, their direct regulatory roles in suppressing the cutaneous inflammatory response have not been revealed or certain members may even promote inflammation [21,22,23,24,25,26,27,28,29]. This review explores how ZNF750, GRHL3, and OVOL1 coordinate epidermal differentiation with immune homeostasis and discusses the potential implications of disruptions in these regulatory networks for inflammatory skin diseases.

## 2. Transcription Factors Driving Terminal Differentiation and Suppressing Inflammation

### 2.1. ZNF750

#### 2.1.1. ZNF750 as a Regulator of Terminal Differentiation

**Figure 1:**
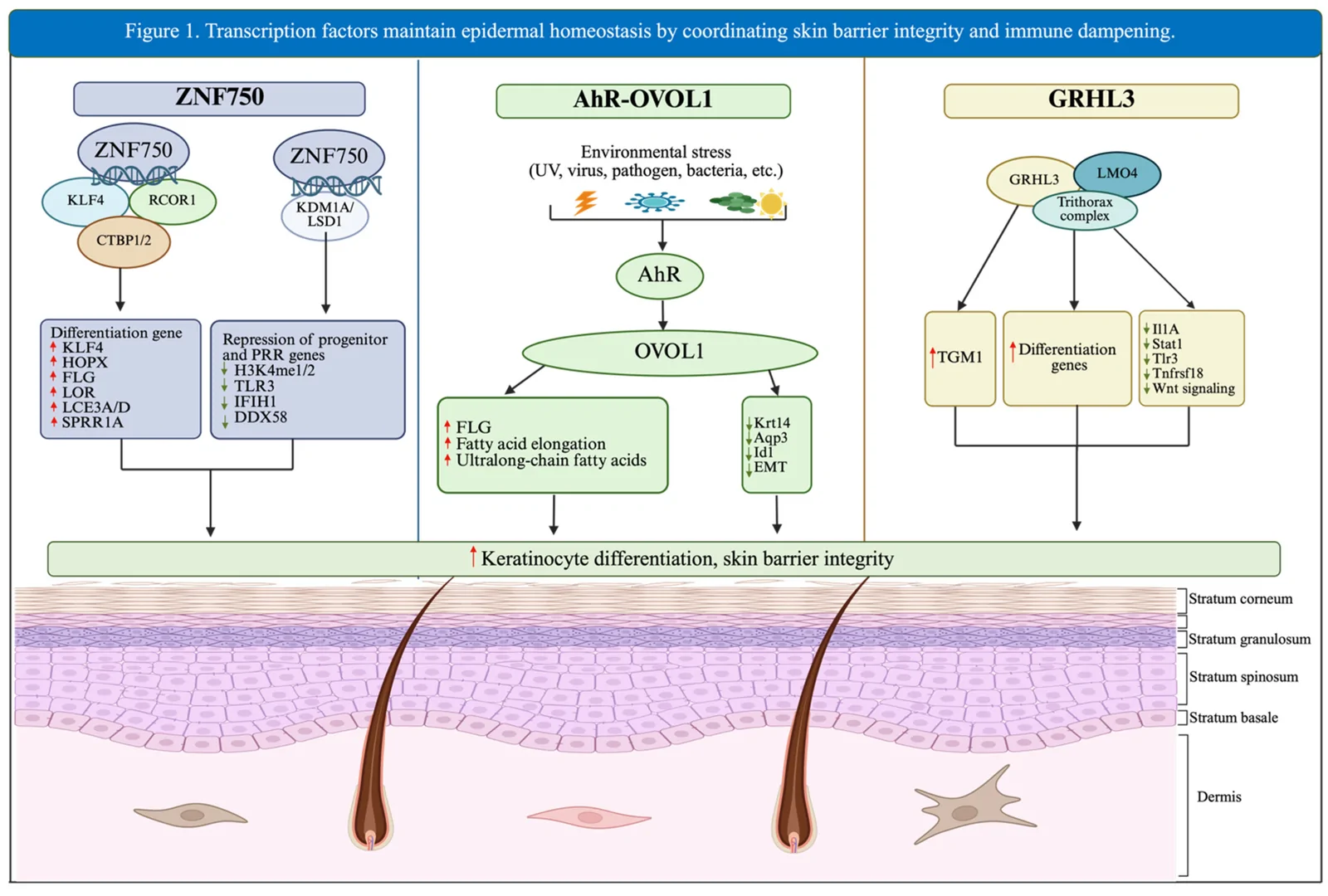
Transcription factors maintain epidermal homeostasis by coordinating skin barrier integrity and immune dampening. Schematic representation of the epidermis illustrating the homeostatic functions of ZNF750/KDM1A, OVOL1/AhR, and GRHL3 in regulating epidermal differentiation, barrier formation, and immune quiescence. Together with KLF4, RCOR1, and CTBP1/2, ZNF750 promotes epidermal differentiation by activating barrier-associated genes, including *KLF4*, *HOPX*, *FLG*, *LOR*, *LCE3A*, *LCE3D*, and *SPRR1*. In complex with KDM1A, ZNF750 epigenetically represses pattern recognition receptors (PRRs), including *TLR3*, *IFIH1*, and *DDX58*, thereby dampening keratinocyte innate immune responses. OVOL1, acting downstream of AhR, promotes epidermal differentiation by enhancing FLG expression, fatty acid elongation, and ultralong-chain fatty acid synthesis, while suppressing proliferation-associated genes (*Krt14*, *Id1*, and *Aqp3*) and inhibiting epithelial–mesenchymal transition (EMT). GRHL3, in cooperation with chromatin-remodeling complexes (trxG and LMO4), suppresses inflammatory mediators, including *Il1a*, *Stat1*, *Tlr3*, *Tnfrsf18*, and components of the Wnt signaling pathway, while promoting *TGM1* expression and keratinocyte differentiation programs. Red arrows indicate activation or increased expression, whereas green arrows indicate repression or decreased expression. Figure created with (https://www.biorender.com).

ZNF750, a member of the C2H2-type zinc finger protein family, is first turned on in the suprabasal layer with maximal expression in the terminal differentiated layers of the epidermis [30]. ZNF750 knockdown in regenerated human skin reduced the expression of late epidermal differentiation markers, including *FLG*, *LOR*, late cornified envelope 3A (*LCE3A*), late cornified envelope 3D (*LCE3D*), and small proline-rich protein 1A (*SPRR1A*). Conversely, ectopic overexpression of ZNF750 in undifferentiated progenitor cells drives terminal differentiation gene expression and generates a mature keratinocyte phenotype, supporting a key role for ZNF750 in epidermal terminal differentiation [30]. Chromatin immunoprecipitation qPCR (ChIP-qPCR) analyses confirmed that ZNF750 co-occupies *p63* target loci and that ZNF750 overexpression in p63-null cells partially restored the differentiation transcriptional program, supporting its role as a downstream effector of p63 [30,31]. ChIP assays in terminally differentiated keratinocytes demonstrated direct binding of ZNF750 to the *KLF4* transcriptional start site [30]. These findings indicate that ZNF750 promotes terminal differentiation, in part, through transcriptional activation of *KLF4*. In addition to being an activator of *KLF4*, ZNF750 also interacts with KLF4, RCOR1, and CTBP1/2 to induce differentiation gene expression [32]. This includes regulation of granular layer gene expression and the expression of lipid-modifying enzymes and structural proteins required for cornified envelope formation [30,32]. HOP homeobox (*HOPX*) has also been identified as a direct transcriptional target of ZNF750, which is involved in late epidermal differentiation [33]. To drive the transition from proliferative basal cells to a post-mitotic state, ZNF750 associates in a separate complex with lysine-specific demethylase 1A (KDM1A), REST corepressor 1 (RCOR1), and C-terminal-binding proteins 1 and 2 (CTBP1/2) to repress progenitor gene expression [32]. These findings suggest that ZNF750 regulates multiple transcriptional programs during epidermal maturation. Thus, ZNF750 is an important regulator of epidermal differentiation, mediating the transition from proliferative progenitors to post-mitotic, barrier-forming keratinocytes (Figure 1). In addition to promoting structural differentiation, ZNF750 also suppresses epidermal innate immune signaling, as discussed below.

#### 2.1.2. ZNF750 in Epidermal Immune Homeostasis and Inflammation

Beyond its fundamental role in facilitating terminal differentiation, ZNF750 also inhibits inflammatory signaling within the epidermis. As the primary cellular constituent of the epidermis, keratinocytes serve as central regulators of cutaneous immune homeostasis. In this context, ZNF750 contributes to the maintenance of immune quiescence in terminally differentiated keratinocytes [14]. Using a tamoxifen-inducible K14-CreER mouse model, the conditional deletion of *Zfp750* (mouse homologue of ZNF750) in the interfollicular epidermis exacerbated UVB-induced inflammation, including erythema, scaling, and neutrophilic infiltration, mimicking psoriasiform dermatitis [14,34]. The ablation of ZNF750 in the interfollicular epidermis also led to lymphadenopathy and upregulation of inflammatory cytokines and pattern recognition receptors (PRRs) at the transcriptional level. PRRs are crucial for cells to recognize pathogens and mount an innate immune response [35]. These findings substantiate that ZNF750 restrains inflammatory signaling under homeostatic conditions [14]. To elucidate the mechanism by which ZNF750 mediates immune suppression, ZNF750-silenced human keratinocytes were stimulated with Poly (I:C), a synthetic double-stranded RNA that activates antiviral signaling pathways. ChIP-qPCR analyses revealed that ZNF750 directly binds to and represses genes encoding PRRs, including *TLR3*, *IFIH1*, and *DDX58* [14]. Even in the absence of Poly (I:C), ZNF750 binds and suppresses the expression of these inflammatory genes in differentiated keratinocytes, thereby supporting its role in maintaining epidermal immune homeostasis. Consistent with this, keratinocytes with silenced *ZNF750* exhibited increased expression of pro-inflammatory mediators and PRRs, including Toll-like receptor 3 (TLR3) and interferon-stimulated genes. ZNF750 silences inflammatory genes by recruiting the histone H3 lysine 4 mono- and dimethyl (H3K4me1/2) demethylase, KDM1A/LSD1, to attenuate inflammatory gene expression (Figure 1). Conditional loss of Kdm1a in the epidermis also led to excessive inflammatory responses, which mimicked inflammatory skin diseases [14,36]. These findings indicate that the ZNF750/KDM1A axis plays a vital role in suppressing inflammatory signaling in the epidermis [14].

To further define the role of TLR3 in this pathway, in both in vitro and in vivo settings, double deletion of *ZNF750* and *TLR3*, followed by Poly (I:C) or UVB radiation, confirmed that ZNF750 suppresses inflammatory signaling, in part, by limiting TLR3 signaling [14]. In summary, as epidermal cells differentiate, they turn on ZNF750 expression, which is then able to recruit KDM1A to suppress inflammatory genes such as PRRs. Since differentiated keratinocytes are found on the outer surface of the skin and constantly interact with the environment, dampening PRR expression prevents unwanted, excessive inflammation. In contrast, if a pathogen or tissue damage occurs that penetrates deeper into the epidermis where the undifferentiated keratinocytes reside, a strong inflammatory response occurs due to the absence of ZNF750 and thus higher levels of PRR expression.

**Figure 2:**
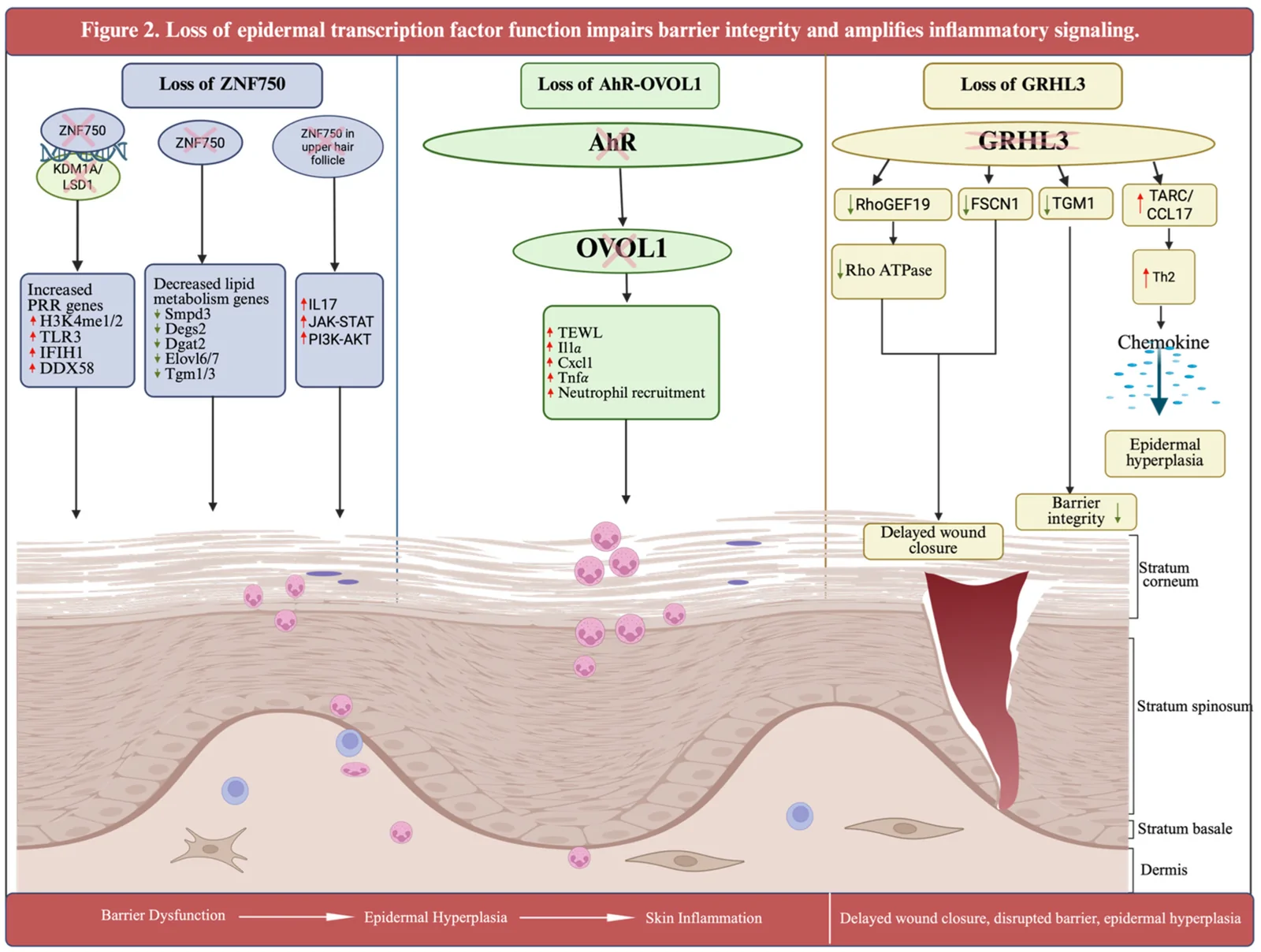
Loss of epidermal transcription factor function impairs barrier integrity and amplifies inflammatory signaling. Schematic representation of the molecular and cellular consequences of ZNF750/KDM1A, OVOL1, and GRHL3 loss of function in the epidermis (red X). Loss of ZNF750/KDM1A leads to increased H3K4me1/2 levels and enhanced expression of pattern recognition receptors (PRRs) including *TLR3*, *IFIH1*, and *DDX58*, while loss of ZNF750 suppresses lipid metabolism- and barrier-associated genes (*Smpd3*, *Degs2*, *Dgat2*, *Elovl6*, *Elovl7*, *Tgm1*, and *Tgm3*). These alterations contribute to epidermal barrier dysfunction, epidermal hyperplasia, and cutaneous inflammation. In the upper hair follicle, ZNF750 deficiency further promotes activation of the JAK–STAT, PI3K–AKT, and IL-17 signaling pathways, resulting in psoriasis-like inflammation. Loss of OVOL1 impairs aryl hydrocarbon receptor (AhR)-mediated epidermal differentiation, resulting in increased transepidermal water loss (TEWL), elevated expression of Il1a, Cxcl1, and Tnf-α, increased neutrophil infiltration, and the development of epidermal hyperplasia and psoriasis-like skin inflammation. Loss of GRHL3 caused delayed wound healing through reduced expression of Rho ATPase, FSCN1, TGM1, and led to impaired barrier integrity. In addition, GRHL3 deficiency promotes epidermal hyperplasia by increasing TARC/CCL17 expression, thereby enhancing chemokine-mediated recruitment of Th2 lymphocytes and amplifying cutaneous inflammatory responses. Red arrows indicate activation or increased expression, whereas green arrows indicate repression or decreased expression. Figure created with (https://www.biorender.com).

A recent study also demonstrated that deletion of *Zfp750* from the upper-hair-follicle of mice resulted in a psoriasis-like phenotype and lymphadenopathy [37]. This was characterized by increased keratin 6 (K6) expression, a marker of epidermal inflammation and hyperproliferation, and elevated immune cell infiltration, including neutrophils and CD4 T lymphocytes, in the skin of *Zfp750*-knockout mice [37]. Bulk RNA sequencing of cells depleted of *Zfp750* from murine skin revealed an increase in IL-17, Janus kinase–signal transducer and activator of transcription (JAK-STAT), and phosphoinositide 3-kinase (PI3K)-AKT signaling pathways [37]. These findings extend the understanding of ZNF750’s role beyond the interfollicular epidermis to follicular immune homeostasis. The data suggest that ZNF750 maintains epidermal immune equilibrium by recruiting KDM1A to suppress innate immune sensor genes. Disruption of the ZNF750/KDM1A regulatory axis is sufficient to predispose to psoriasiform inflammation, thereby indicating that ZNF750 contributes to both barrier formation and the suppression of epidermal immune activation (Figure 2). In addition to its roles in differentiation and immunosuppression, ZNF750 also regulates epidermal lipid metabolism, as discussed below.

#### 2.1.3. ZNF750 in Epidermal Lipid Metabolism

Beyond its roles in epidermal differentiation and innate immunity, ZNF750 directly influences lipid metabolism, thereby linking transcriptional regulation to the biochemical composition and permeability of the stratum corneum [38]. This protective barrier depends on an intercellular lipid matrix comprising ceramides, glucosylceramides, and glycosphingolipids to prevent water and electrolyte loss. Disruption of this lipid matrix undermines barrier integrity and contributes to inflammatory skin diseases [39]. Butera et al. reported that ZNF750 was associated with skin lipid metabolism through lipidomic analysis and studies involving constitutive global *Zfp750* knockout mice [38]. These mice experienced perinatal lethality due to skin barrier failure, as evidenced by toluidine blue dye exclusion. Neonates with the knockout exhibited decreased expression of transglutaminase 1 and 3 (*Tgm1* and *Tgm3*), enzymes essential for cross-linking the cornified envelope, alongside increased Ivl, which likely represents a compensatory response to barrier failure [38]. Lipidomic analysis demonstrated an almost complete loss of ceramides and glycosphingolipids within the stratum corneum. Transcriptomic analysis indicated downregulation of lipid metabolic genes involved in ceramide synthesis and fatty acid elongation, including *Sptlc1*, *Kdsr*, *Degs1/2*, *Smpd1-3*, and *Elovl6/7*. ChIP assays confirmed the direct binding of Zfp750 to multiple lipid metabolic genes such as *Degs2*, *Dgat2*, *Smpd3*, *Elovl6*, and *Elovl7*, thus supporting a direct role for Zfp750 in its regulation [38]. These observations were corroborated by a skin-specific *Zfp750* knockout model using K14-Cre, which also exhibited severe barrier defects, postnatal lethality, disrupted epidermal stratification, and dysregulated expression of lipid-processing genes, thereby emphasizing the significance of ZNF750-dependent lipid regulation in maintaining postnatal epidermal homeostasis [40].

Together, these findings show that ZNF750 coordinates multiple aspects of epidermal homeostasis, including differentiation, immune suppression, and lipid metabolism. Therefore, ZNF750 maintains barrier integrity by activating structural genes and regulating lipid metabolism. This offers a mechanistic explanation for how the loss of ZNF750 simultaneously compromises the barrier and increases susceptibility to inflammation (Table 1).

### 2.2. GRHL3

#### 2.2.1. GRHL3’s Role in Barrier Formation

GRHL3 is a key transcriptional regulator of epidermal differentiation, barrier formation, and tissue repair. Early studies in *Grhl3*-null mice revealed severe barrier defects, including loss of intracellular lamellar bodies, weakened stratum corneum membranes, and hyperkeratosis [41]. Conditional deletion of *Grhl3* in postnatal epidermis further showed that *Grhl3* is required to restrain injury-induced epidermal hyperplasia [42]. Transgenic expression of Grhl3 in *Grhl3*-deleted mice can rescue neurulation and skin barrier abnormalities, allowing survival into adulthood [43]. Together, these findings establish GRHL3 as an essential regulator of epidermal barrier integrity during both development and postnatal tissue maintenance.

At the molecular level, GRHL3 regulates epidermal differentiation through conserved transcriptional programs. GRHL3 collaborates with chromatin-remodeling complexes, including the trithorax group complex, to facilitate differentiation of epidermal progenitors [44], and interacts with cofactors such as LMO4 to orchestrate cell migration [45] (Figure 1).

GRHL3 also directly regulates transglutaminase 1, an enzyme essential for cornified envelope formation and epidermal barrier integrity [46]. Overall, GRHL3 integrates differentiation and barrier repair through coordinated transcriptional and chromatin-regulatory mechanisms.

#### 2.2.2. GRHL3 in Wound Healing

GRHL3 also plays a significant role in wound healing, wherein keratinocytes must coordinate polarity, cytoskeletal remodeling, cell migration, and intercellular adhesion. A central mechanism involves the Grhl3–RhoGEF19–RhoA signaling axis. Grhl3 directly regulates RhoGEF19, which activates RhoA GTPase signaling and promotes cytoskeletal organization during epithelial wound closure [47,48]. The loss of *Grhl3* results in decreased *RhoGEF19* expression, attenuates *RhoA* activation, and impairs wound closure; conversely, re-expression of *RhoGEF19* can partially ameliorate the migratory defect observed in *Grhl3*-deficient keratinocytes [48] (Figure 2). This pathway is of particular importance, as RhoA activity supports planar cell polarity in leader cells at the wound margin and facilitates the formation of the actomyosin purse string, a contractile structure that coordinates epithelial sheet migration [49]. Through this mechanism, Grhl3 connects epidermal differentiation status to the cytoskeletal machinery essential for efficient re-epithelialization.

GRHL3 deficiency also disrupts the balance between adhesion and motility. Loss of Grhl3 results in decreased expression of FSCN1, an actin-bundling protein that stabilizes cellular protrusions, while compensatory upregulation of E-cadherin enhances intercellular adhesion [50]. Although increased adhesion may contribute to maintaining epithelial cohesion, excessive adhesion can restrict cytoskeletal flexibility and directional migration. This imbalance likely contributes to the delayed wound closure observed in *Grhl3*-deficient skin. These findings suggest that GRHL3 facilitates wound healing by coordinating keratinocyte migration, cytoskeletal remodeling, polarity, and barrier restoration (Table 1).

#### 2.2.3. GRHL3 in Inflammation and Stem Cell Regulation

In addition to its roles in barrier repair and wound healing, GRHL3 also restrains epidermal inflammation and maintains stem cell homeostasis. Unlike its essential, constitutive role during embryonic skin development, GRHL3 is expressed at low level in the unchallenged adult mouse epidermis and is dispensable for adult skin homeostasis at baseline. GRHL3 is induced in response to epidermal damage, including the immune-mediated damage characteristic of psoriasis: human psoriatic skin shows GRHL3 upregulation that positively correlates with IL-17 activity [42]. This pattern reflects an inducible repair response. GRHL3 expression and chromatin binding increase following mechanical, chemical, or immune-mediated barrier injury, and conditional epidermal deletion of Grhl3 produces an exacerbated damage response, delayed lesion resolution, and increased susceptibility to imiquimod-induced psoriasiform disease, including enhanced CD45+ immune cell infiltration [42,47]. Critically, GRHL3 elevation in human psoriasis is not merely correlative with disease activity but is reversible with treatment. Anti-TNF, anti-IL-23, and anti-IL-17R therapies all reduce GRHL3 expression toward nonlesional levels following treatment [42]. This treatment responsive pattern suggests that GRHL3 upregulation is downstream of the inflammatory cytokine drive rather than a contributor to it, consistent with a repair response. This is a relationship the original authors explicitly compare to inducible tumor-suppressor pathways that are upregulated in response to pathological stress yet can be overwhelmed by a sufficiently strong driving signal [42].

Mechanistically, GRHL3 helps restrain epidermal inflammation by maintaining barrier integrity and limiting immune-amplifying signals. Barrier disruption caused by GRHL3 deletion increases expression of TARC/CCL17, a chemokine that promotes Th2 lymphocyte recruitment and contributes to epidermal hyperplasia [51] (Figure 2). This repressive function is corroborated by genome-wide ChIP-seq data: in imiquimod-treated epidermis, Grhl3 directly binds and suppresses a subset of alarmin and proinflammatory genes, including *Il1a*, *Stat1*, *Tlr3*, *Tnfrsf18*, and *Irf6*. Furthermore, loss of Grhl3 is associated with enhanced immune cell infiltration under low-dose imiquimod challenge, directly linking GRHL3’s repressive chromatin occupancy to restrained immune activation in vivo [42].

GRHL3 also modulates the behavior of epidermal stem and progenitor cells. Single-cell RNA sequencing of the interfollicular epidermis in newborn mice demonstrated that epidermal differentiation proceeds as a gradual continuum rather than a strictly linear process [52]. Within this differentiation landscape, Grhl3 constrains stem cell proliferation partly by repressing components of Wnt signaling, including Wnt4, Lef1, Ctnnb1, Gpc3, and Gsk3b [52]. By promoting differentiation while limiting Wnt-driven self-renewal, GRHL3 helps maintain the balance between epidermal renewal and stem cell expansion. Disruption of this balance may contribute to barrier defects, inflammatory activation, and abnormal keratinocyte proliferation in hyperproliferative skin diseases such as psoriasis. Therefore, GRHL3 appears to restrain epidermal disease through these complementary mechanisms, including limiting immune-amplifying chemokine programs and restricting Wnt-associated stem/progenitor expansion.

### 2.3. OVOL1

#### 2.3.1. OVOL1 in Environmental Sensing and Epidermal Homeostasis

OVOL1 is a transcription factor that plays significant roles in epidermal homeostasis, epithelial differentiation, and environmental sensing. In the skin, OVOL1 is expressed in the differentiated layers of the epidermis and regulates genes in the epidermal differentiation complex (EDC), where it facilitates keratinocyte differentiation and preserves epidermal identity [53,54,55]. A key upstream regulator of OVOL1 is the aryl hydrocarbon receptor (AhR), a ligand-activated transcription factor that enables keratinocytes to respond effectively to xenobiotic environmental stimuli. Upon AhR activation, Ovol1 expression is induced, resulting in suppression of proliferative programs, including Krt14, Id1, Aqp3, and c-Myc, while simultaneously promoting terminal differentiation [56,57]. Through this mechanism, OVOL1 effectively connects environmental sensing with the transcriptional programs essential for maintaining epidermal homeostasis.

In addition to regulating differentiation, OVOL1 directly contributes to epidermal barrier formation. AhR-mediated induction of OVOL1 promotes the expression of filaggrin (FLG), a crucial structural component of the cornified envelope that is vital for effective epidermal barrier function [58]. Moreover, AhR–OVOL1 signaling has been associated with epidermal lipid metabolism by regulating fatty acid elongation and the synthesis of ultralong-chain fatty acids, which are required for ω-O-acylceramide production and water retention [57,59]. These findings indicate that OVOL1 orchestrates both the structural and lipid components of the epidermal barrier, thereby supporting barrier integrity and maintaining cutaneous homeostasis.

The significance of OVOL1 in preserving epithelial identity becomes particularly apparent when considered in conjunction with its paralog, OVOL2. Although OVOL1 can function independently, the two factors exhibit partially overlapping roles in epithelial tissues. The combined deletion of Ovol1 and Ovol2 results in severe defects in keratinocyte differentiation, cell adhesion, and epidermal barrier formation, culminating in perinatal lethality [57,60]. Similar anomalies in barrier function are observed following inducible deletion of both genes in adult skin, indicating that OVOL1 and OVOL2 are essential not only for epidermal development but also for the maintenance of adult epidermal homeostasis [60]. Mechanistically, OVOL1 and OVOL2 uphold epithelial identity by repressing transcriptional programs associated with epithelial–mesenchymal transition (EMT). The loss of both factors increases EMT-related gene expression and disrupts cell adhesion pathways, thereby compromising epithelial integrity and barrier function [59,60]. Comprehensive ChIP-seq analyses further confirmed that OVOL1 and OVOL2 co-occupy regulatory regions controlling extensive networks involved in differentiation, cell adhesion, EMT suppression, and inflammatory regulation [60]. This suggests that OVOL proteins act as pivotal regulators that sustain epidermal identity by promoting differentiation while inhibiting transcriptional programs linked to epithelial plasticity and inflammation. In addition to their functions within the epidermis, OVOL1-dependent AhR signaling also plays a role in hair follicle biology. Evidence from pharmacological AhR activation studies suggests that OVOL1 functions downstream of AhR to support hair growth and follicular homeostasis [61,62]. Thus, OVOL1 appears to integrate environmental signals with differentiation programs across multiple epithelial compartments of the skin.

By integrating AhR-dependent environmental responses with processes such as differentiation, barrier formation, lipid metabolism, and the maintenance of epithelial identity, OVOL1 plays a crucial role in safeguarding skin integrity under both physiological and environmental stress conditions (Figure 1). The degree to which disruption of these functions may contribute to inflammatory skin diseases will be examined in the subsequent section.

#### 2.3.2. OVOL1 in Inflammation

In addition to its role in epidermal differentiation and barrier formation, OVOL1 has been recognized as an essential regulator of cutaneous inflammatory homeostasis. In murine atopic dermatitis models, treatment of Ovol1-deleted skin with MC903 led to increased epidermal thickness, transepidermal water loss (TEWL), and immune cell infiltration as compared to control mice [57]. Ovol1 was found to directly bind and repress *Id1*. Small molecule inhibition of Id1 led to amelioration of the Ovol1 loss of function phenotype which resulted in a decrease in epidermal thickness, TEWL, and neutrophil recruitment [57]. The anti-inflammatory properties of OVOL1 are further illustrated in models of epidermal inflammation related to psoriasis [55]. *Ovol1*-deficient mice exhibit heightened susceptibility to imiquimod-induced psoriasis-like conditions, characterized by impaired barrier function, elevated expression of proinflammatory mediators such as *Il1a*, and increased infiltration of neutrophils [55]. These findings suggest that disruption of OVOL1-dependent barrier mechanisms promotes the activation of inflammatory pathways that enhance cutaneous immune responses.

At the cellular level, OVOL1 deficiency modifies epidermal differentiation dynamics. Single-cell RNA sequencing analyses of inflamed epidermis revealed elevated basal keratinocyte proliferation, dysregulated early differentiation, and disruption of the normal transition from basal to mature spinous cell states [55]. These observations suggest that OVOL1 not only facilitates terminal differentiation but also helps maintain the orderly progression of epidermal cell maturation necessary for tissue homeostasis. In line with these phenotypic changes, Ovol1-deficient skin demonstrates increased expression of inflammatory and metabolic gene signatures, including pathways associated with TNF-α and cytokines [60]. By preserving barrier integrity, suppressing abnormal keratinocyte proliferation, and limiting cytokine- and neutrophil-associated inflammatory responses, OVOL1 appears to serve as a critical safeguard against chronic inflammatory skin diseases.

Although ZNF750, GRHL3, and OVOL1 operate through distinct regulatory pathways, they converge upon a common epidermal principle: that terminal differentiation is associated with the repression of inflammatory and stress-related programs. ZNF750 promotes differentiation and silencing of innate immune sensors. GRHL3 orchestrates stress-responsive barrier repair, cytoskeletal remodeling, and the inhibition of chemokine and Wnt-related hyperproliferative processes. OVOL1 integrates environmental AhR signaling with barrier gene expression and the suppression of proliferative and inflammatory pathways. Therefore, inflammatory skin diseases may result not only from immune cell dysregulation but also from the failure of keratinocyte-intrinsic transcriptional networks that normally uphold barrier function and immune quiescence (Figure 2 and Table 1).

## 3. Clinical Relevance of ZNF750, GRHL3, and OVOL1 in Skin Diseases

**Figure 3:**
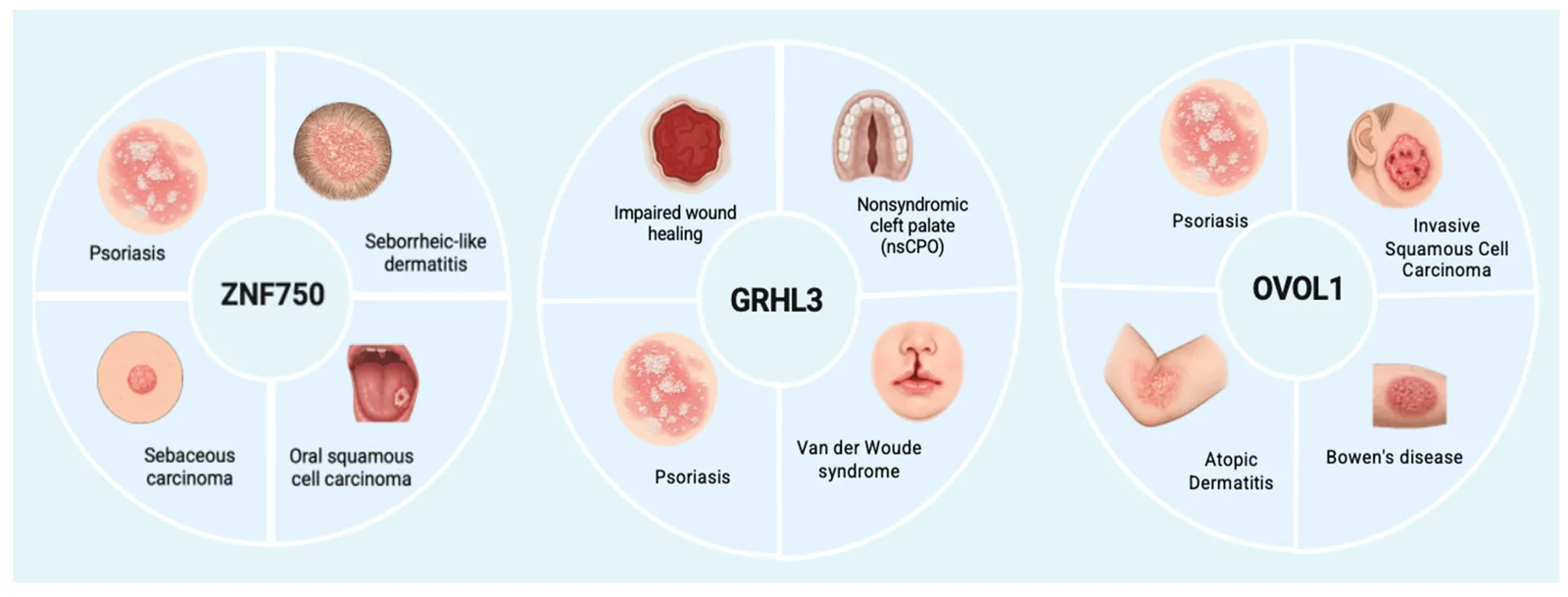
Dysregulation of epidermal transcription factors in human diseases. Wheel diagrams summarizing disease associations for ZNF750, GRHL3, and OVOL1. *ZNF750* dysregulation is linked to inflammatory skin conditions (psoriasis, seborrheic-like dermatitis) and malignancies (oral squamous cell carcinoma, sebaceous carcinoma). Pathogenic variants in *GRHL3* are associated with impaired wound healing, nonsyndromic cleft palate (nsCPO), Van der Woude syndrome, and psoriasis. The *OVOL1* locus has been identified in GWAS as a susceptibility locus for atopic dermatitis. Its expression is downregulated in psoriasis, Bowen’s disease, and invasive cutaneous squamous cell carcinoma (cSCC). Clinical cartoon illustrations are schematic and do not represent actual patient photographs. Figure created with BioRender (https://www.biorender.com) and ChatGPT (OpenAI).

Extensive genetic, transcriptomic, and histopathological evidence indicates that dysregulation of epidermal differentiation-associated transcription factors contributes to a wide range of human diseases, including chronic inflammatory skin disorders, developmental epithelial abnormalities, and cutaneous malignancies. While dysregulation of ZNF750 and OVOL1 have stronger links to inflammatory skin diseases such as atopic dermatitis or psoriasis, GRHL3 has broader clinical relevance to developmental epithelial disorders and wound healing (Figure 3). Collectively, research on these factors supports the notion that transcriptional programs controlling epidermal differentiation are linked to barrier integrity, immune homeostasis, and sustained tissue maintenance.

### 3.1. Inflammatory Skin Diseases

Among the transcription factors discussed in this review, ZNF750 and OVOL1 have been most extensively associated with inflammatory skin disease. Clinical evidence supports a role for ZNF750 in psoriasis-related disorders. Pathogenic ZNF750 mutations were initially identified in patients presenting with seborrheic dermatitis-like disease with psoriasiform features, characterized by erythematous, scaly skin lesions [63]. Subsequent genetic studies linked ZNF750 mutations and reduced expression to familial and sporadic forms of psoriasis, further supporting its role in maintaining epidermal homeostasis and preventing disease [64,65].

OVOL1 has also emerged as a clinically relevant regulator of inflammatory skin disease. Genome-wide association studies (GWAS) have identified OVOL1 as a susceptibility locus for atopic dermatitis, implicating OVOL1-dependent pathways in inflammatory skin conditions [66]. Consistent with experimental findings, skin biopsies from patients with atopic dermatitis show alterations in components of the AhR–OVOL1–ID1 signaling axis, supporting the clinical relevance of this pathway in human disease [57]. Given that AhR–OVOL1 signaling regulates epidermal differentiation, barrier formation, and immune homeostasis, disruption of this pathway may contribute to the barrier dysfunction and chronic inflammation characteristic of atopic dermatitis [57]. Evidence also links OVOL1 to psoriasis. Expression of both OVOL1 and filaggrin progressively decreases from normal skin to peri-lesional and fully developed psoriatic lesions, and their expression levels negatively correlate with Psoriasis Area and Severity Index (PASI) scores [67] (Figure 3).

### 3.2. Cutaneous Malignancies

In addition to inflammatory disorders, increasing evidence indicates that differentiation-associated transcription factors function as tumor suppressors in epithelial cancers. ZNF750 is the most well-characterized example and has been implicated in several squamous malignancies. Mutations and diminished expression of ZNF750 have been documented in sebaceous carcinoma, esophageal squamous cell carcinoma, and other epithelial cancers, with transcriptomic and single-cell RNA sequencing analyses consistently demonstrating loss of ZNF750-associated differentiation programs [68,69,70,71]. Functional studies further corroborate a tumor-suppressive role, as restoration of ZNF750 expression decreases metastatic behavior in oral squamous cell carcinoma cells [72]. These findings imply that the loss of ZNF750 contributes to malignant progression through the disruption of epithelial differentiation and maintenance mechanisms.

Similarly, OVOL1 has been implicated in cutaneous tumorigenesis. OVOL1 expression decreases during progression from Bowen’s disease to invasive squamous cell carcinoma, potentially through dysregulation of c-Myc-dependent pathways [56]. These observations suggest that OVOL1 contributes to the preservation of epithelial identity and may suppress pathways associated with malignant transformation and disease progression (Figure 3).

### 3.3. Developmental Epithelial Disorders

The clinical significance of GRHL3 extends beyond inflammatory skin conditions and neoplasms to encompass developmental disorders affecting epithelial tissues. Variants in GRHL3 have been linked to nonsyndromic cleft palate and Van der Woude syndrome, thereby underscoring the importance of GRHL3-dependent transcriptional programs in craniofacial and epithelial development [73,74,75]. These developmental phenotypes are consistent with experimental studies demonstrating the critical roles of GRHL3 in epithelial differentiation, cell migration, wound healing, barrier formation, and tissue morphogenesis. The link between GRHL3 and both developmental anomalies and postnatal barrier dysfunction further emphasizes its pivotal role in maintaining epithelial integrity throughout the lifespan (Figure 3).

## 4. Therapeutic Implications

The transcriptional networks governing epidermal differentiation, barrier integrity, and immune homeostasis discussed throughout this review represent promising therapeutic targets for inflammatory skin diseases. Traditionally, therapeutic development in dermatology has primarily focused on suppressing immune cell activity and inflammatory cytokine signaling. However, growing evidence suggests that restoring keratinocyte-intrinsic differentiation programs may offer an additional strategy to correct the fundamental barrier dysfunction and immune dysregulation characteristic of disorders such as psoriasis and atopic dermatitis. Among the pathways examined in this review, the aryl hydrocarbon receptor (AhR) signaling pathway provides the most compelling example of successful clinical translation [76]. Tapinarof, a topical AhR agonist, received FDA approval for the treatment of plaque psoriasis in 2022 and atopic dermatitis in 2024 [77]. Clinical efficacy was demonstrated in the Phase III PSOARING 1 and PSOARING 2 trials for psoriasis [78,79,80] and in the ADORING 1 and ADORING 2 trials for atopic dermatitis [81], which showed significant improvements in disease severity and durable treatment responses extending beyond the treatment period in patients with psoriasis. Although the exact molecular mechanisms underpinning Tapinarof activity remain incompletely understood [77], its clinical success strongly underscores the therapeutic significance of AhR-dependent transcriptional pathways in skin disease. Experimental studies have demonstrated that AhR activation promotes OVOL1-dependent epidermal differentiation and filaggrin expression and suppresses inflammatory signaling [57,58]. These findings offer proof of concept that targeting differentiation-associated transcriptional networks can yield meaningful therapeutic benefit. The clinical efficacy of cytokine-targeted therapies also underscores the importance of epidermal differentiation programs. In psoriasis, treatment with anti-TNF, anti-IL-23, and anti-IL-17R therapies correlates with decreased GRHL3 expression, likely indicative of normalization of stress-induced epidermal repair mechanisms following the resolution of inflammation [30,37]. These findings highlight the reciprocal relationship between inflammatory signaling pathways and differentiation-associated transcriptional networks and imply that restoring epidermal homeostasis may contribute to therapeutic responses.

Beyond currently approved therapies, transcription factors such as ZNF750, GRHL3, and OVOL1 are recognized as potential therapeutic targets due to their central roles within regulatory networks governing differentiation, barrier formation, proliferation, and inflammatory signaling. Modulating these pathways could theoretically address multiple pathogenic processes simultaneously. These findings support an emerging therapeutic paradigm in which epidermal differentiation is regarded not merely as a structural process but as an active regulator of immune homeostasis. Future therapeutic strategies may therefore integrate immune-targeted interventions with approaches to restore keratinocyte differentiation and barrier integrity, thereby offering a more comprehensive treatment for inflammatory and barrier-deficient skin disorders.

## 5. Future Directions

Although substantial progress has been achieved in delineating the roles of ZNF750, GRHL3, and OVOL1 in epidermal differentiation and inflammatory regulation, several critical questions remain unsettled. A significant gap pertains to the relationship between these transcription factors and epidermal lipid metabolism. While ZNF750 has been associated with lipid homeostasis through integrated transcriptomic and lipidomic analyses, the extent to which GRHL3 and OVOL1 influence epidermal lipid synthesis, ceramide metabolism, and barrier-associated lipid pathways necessitates further examination. Considering the pivotal role of lipid abnormalities in inflammatory skin diseases, further research into these mechanisms may uncover vital therapeutic opportunities.

Another area requiring further study is the interaction between differentiation-associated transcription factors and epigenetic regulatory machinery. Current evidence demonstrates that ZNF750 cooperates with KDM1A/LSD1 to repress inflammatory gene programs, whereas GRHL3 interacts with chromatin-remodeling complexes to regulate differentiation and tissue repair. However, the broader epigenetic networks governing transcription factor activity in the epidermis remain incompletely characterized. Defining how these factors interact with chromatin modifiers, enhancer landscapes, and cytokine-responsive regulatory elements may uncover shared regulatory mechanisms linking barrier dysfunction to chronic inflammation. Additionally, epigenetic regulators represent druggable targets, which traditionally has not been the case with transcription factors.

Emerging gene-modifying technologies may offer potential opportunities for therapeutic interventions. Notably, CRISPR activation (CRISPRa) systems employing catalytically inactive Cas9 (dCas9) fused to transcriptional activators such as VP64 or VPR present a prospective strategy for re-establishing physiological expression levels of transcription factors that are diminished due to mutation, haploinsufficiency, or disease-related repression [82,83]. Unlike traditional gene-replacement methods, CRISPRa facilitates locus-specific transcriptional activation without causing double-strand DNA breaks, thereby potentially maintaining endogenous regulatory mechanisms. Nevertheless, the utilization of CRISPRa in the treatment of inflammatory skin diseases remains largely speculative. Significant challenges concerning target validation, delivery mechanisms, specificity, durability, and safety must be addressed prior to considering these types of therapeutic applications. Consequently, rather than being viewed as an immediately viable therapeutic option, CRISPRa should currently be regarded as a distant conceptual prospect for the restoration of the epidermal differentiation processes.

## 6. Conclusions

ZNF750, GRHL3, and OVOL1 exemplify a broader class of epidermal transcription factors that coordinate terminal differentiation with suppression of inflammatory responses. Although these factors are activated through distinct developmental, environmental, and stress-responsive pathways, they converge on a common biological principle: maintenance of epidermal homeostasis requires active transcriptional programs that simultaneously promote barrier formation and restrain inflammation.

Disruptions in these regulatory networks, caused by mutations, reduced expression, or environmental factors, can undermine barrier function, enhance inflammatory signaling, and increase susceptibility to chronic skin diseases.

Clinical and experimental evidence increasingly supports the concept that inflammatory skin disorders arise not only from dysregulated immune responses but also from failure of keratinocyte-intrinsic transcriptional programs that preserve epithelial identity and barrier competence. The clinical success of tapinarof provides proof of concept that targeting differentiation-associated transcriptional pathways can yield meaningful therapeutic benefit and highlights the translational relevance of the mechanisms discussed throughout this review.

This supports a model in which epidermal differentiation factors serve as key regulators of barrier integrity, immune homeostasis, and disease susceptibility. Further research into these transcriptional networks and their downstream pathways will enhance our understanding of inflammatory skin diseases and may ultimately facilitate the development of therapies to restore epidermal function by re-establishing the regulatory programs that sustain cutaneous homeostasis.

## 7. The Limitations of This Review

A limitation of this review is its intentionally narrow focus, concentrating on ZNF750, GRHL3, and OVOL1 based on evidence of epidermal gain- or loss-of-function inflammatory phenotypes, direct binding to inflammatory genes, and transcriptional repression of these targets. Consequently, other epidermal transcription factors that regulate inflammation indirectly, primarily activate inflammatory genes, or lack sufficient mechanistic evidence were not included. Therefore, this review does not encompass the full complexity of transcription factor regulation of epidermal inflammation.

## Figures and Tables

**Table 1:** Comparison between ZNF750, GRHL3, and OVOL1 transcription factors.

Transcription Factor	Main Epidermal Function	Upstream Regulator	Cofactors	Downstream Targets and Downregulated Genes	Immune/Inflammatory Mechanism	Disease Relevance	Therapeutic Implication	Mouse Types	Mouse Phenotype
ZNF750	- Terminal differentiation - Epidermal lipid metabolism - Barrier formation	p63	- KDM1A - RCOR1 - CTBP1/2 - KLF4	- KLF4 - HOPX - Differentiation genes (FLG, LOR, LCE3A/D, SPRR1A) - Lipid metabolism genes (Degs2, Dgat2, Elovl6/7, Smpd3, Tgm1/3) - Repression of PRR-associated genes, including TLR3, IFIH`, DDX58	- Epigenetic silencing of innate immune sensors via KDM1A/LSD1, H3K4me1/2	- Psoriasis - Seborrheic-like dermatitis - Oral and esophageal squamous cell carcinoma - Sebaceous carcinoma	Potential future application of CRISPRa-mediated restoration of ZNF750 expression.	- Epidermal-specific *Zfp750* loss - Upper hair follicle-specific *Zfp750* loss	- Barrier defects, dysregulated lipid metabolism - Psoriasiform inflammation
GRHL3	- Stress-induced barrier repair - Wound closure - Cytoskeletal remodeling - Keratinocyte migration - Stem cell restraint		- trxG - LMO4	- Transglutaminase 1 - RhoGEF19 - FSCN1 - Wnt pathway-associated genes	- Suppresses Th2-associated inflammation via CCL17/TARC regulation - Limits Wnt-driven keratinocyte hyperproliferation	- Impaired wound healing - Nonsyndromic cleft palate with oral adhesions (nsCPO) - Van der Woude syndrome - Psoriasis	Indirect modulation through cytokine-targeted anti-inflammatory therapies	Epidermal-specific *Grhl3* loss	- Epidermal hyperplasia - Impaired wound closure - Barrier defects - Developmental epithelial abnormalities
OVOL1	- Epidermal differentiation; hair follicle biology - Environmental sensing - EMT suppression - Barrier maintenance	AhR		- Flg, Id1, Aqp3, Krt14 - c-Myc-associated differentiation pathways	- Decreases TEWL - Decreases Il1a, Cxcl1, Tnf-α	- Atopic dermatitis (GWAS locus) - Psoriasis - Bowen’s disease - Invasive SCC	AhR agonists (e.g., tapinarof) and modulation of AhR–OVOL1 signaling.	*Ovol1*/*Ovol2* double knockout	- Barrier defects - Upregulation of EMT-associated gene programs - Perinatal lethality

## Data Availability

No new data were created or analyzed in this study. Data sharing is not applicable to this article.

## Abbreviation

AhR	Aryl Hydrocarbon Receptor
Aqp3	Aquaporin 3
CALML5	Calmodulin-like 5
CCL17 (TARC)	C-C Motif Chemokine Ligand 17 (Thymus and Activation Regulated Chemokine)
ChIP	Chromatin Immunoprecipitation
ChIP-seq	Chromatin Immunoprecipitation Sequencing
CLDN1	Claudin 1
c-Myc	Cellular myelocytomatosis oncogene
CTBP1/2	C-terminal Binding Protein 1/2
CRISPRa	CRISPR Activation
dCas9	Catalytically Dead Cas9
DDX58	DExD/H-Box Helicase 58 (RIG-I)
DEGS2	Delta (4)-Desaturase, Sphingolipid 2
DGAT2	Diacylglycerol O-Acyltransferase 2
dsRNA	Double-Stranded RNA
EDC	Epidermal Differentiation Complex
ELOVL6/ELOVL7	Elongation of Very Long Chain Fatty Acids Protein 6/7
EMT	Epithelial–Mesenchymal Transition
FDA	Food and Drug Administration
FLG	Filaggrin
GRHL3	Grainyhead-like 3
GWAS	Genome-Wide Association Study
HOPX	HOP Homeobox
H3K4	Histone 3 Lysine 4 (histone modification mark)
ID1	Inhibitor of DNA Binding 1
IFE	Interfollicular Epidermis
IFN	Interferon
IFIH1	Interferon Induced with Helicase C Domain 1
IL-17	Interleukin 17
IVL	Involucrin
JAK-STAT	Janus Kinase–Signal Transducer and Activator of Transcription
KLF4	Kruppel-like Factor 4
Krt14	Keratin 14
K14-Cre/K14CreER	Keratin 14 promoter driven Cre recombinase/inducible version
KDM1A	Lysine-Specific Histone Demethylase 1A (also known as LSD1)
KDSR	3-Ketodihydrosphingosine Reductase
LCE3A/LCE3D	Late Cornified Envelope 3A/3D
LMO4	LIM Domain Only 4
LOR	Loricrin
LSD1	Lysine-Specific Demethylase 1 (alias of KDM1A)
nsCPO	Nonsyndromic Cleft Palate with Oral Adhesions
OVOL1/Ovol1	Ovo-like zinc finger 1 (human/mouse ortholog)
PASI	Psoriasis Area and Severity Index
p63	Tumor Protein 63
PI3K-AKT	Phosphoinositide 3-Kinase–Protein Kinase B Signaling Pathway
Poly(I:C)	Polyinosinic:Polycytidylic acid
PRRs	Pattern Recognition Receptors
QPCR	Quantitative Polymerase Chain Reaction
RCOR1	REST Corepressor 1
RhoA	Ras Homolog Family Member A
RhoGEF19	Rho Guanine Nucleotide Exchange Factor 19
RIG-I	Retinoic Acid-Inducible Gene I
RNA-seq	RNA Sequencing
ScRNA-seq	Single-cell RNA Sequencing
SC	Stratum corneum
SCC	Squamous Cell Carcinoma
SMPD1-3	Sphingomyelin phosphodiesterase 1-3
SPRR1A	Small Proline-Rich Protein 1A
*SPTLC1*	Serine palmitoyltransferase long chain base subunit 1
TEWL	Transepidermal Water Loss
TGM1	Transglutaminase 1
TGM3	Transglutaminase 3
TLR3	Toll-Like Receptor 3
TNF-α	Tumor Necrosis Factor Alpha
trxG	Trithorax Group
TSS	Transcription Start Site
UVB	Ultraviolet B
VPR	VP64-p65-Rta transcriptional activator
VP64	Tetrameric VP16 activation domain
Wnt signaling	Wingless-related integration site signaling pathway
Zfp750	Zinc Finger Protein 750 (mouse ortholog)
ZNF750	Zinc Finger Protein 750
